# Swedish journalists' perceptions of legal protection against unlawful online harassment

**DOI:** 10.3389/fsoc.2023.1154495

**Published:** 2023-04-17

**Authors:** Oscar Björkenfeldt

**Affiliations:** Sociology of Law Department, Lund University, Lund, Sweden

**Keywords:** online harassment, journalism, legal protection and enforcement, norms and attitudes, normalization, free speech, public discourse, social control

## Abstract

This study examined journalists' perceptions regarding the legal system's ability to protect them against online harassment. By utilizing open-ended survey responses from respondents with varying levels of trust in the legal system, the findings suggested a need for increased technical proficiency, resources, and priority within the legal system to adequately address the issue. Additionally, a reciprocal relationship between the normalization of online harassment within the journalistic profession and the legal system's commitment to providing protection was identified. However, the study also found that when the legal system's mediated approach to online harassment is positive, it affects attitudes and norms relating to legal protection. Consequently, it reveals a unique insight into how journalists respond to the message conveyed by fair treatment and respect from the legal system. Notably, this result implies that when such messages are internalized, journalists feel more empowered to take measures against online harassment. As a result of this analysis, I propose that current laws should be implemented more effectively and that policy strategies should be developed to positively influence social norms and social control to bolster journalistic autonomy and freedom of speech in the digital age.

## Introduction

In recent years, a growing amount of literature has been published on journalist exposure to online harassment in countries such as Sweden (Löfgren Nilsson and Örnebring, 2016), Switzerland (Stahel and Schoen, 2020), the UK (Binns, 2017), Australia (North, 2016), the United States (Lewis et al., 2020), and Taiwan (Chen et al., 2018), to name a few. This research has established that online harassment has detrimental consequences for journalists' wellbeing (Löfgren Nilsson and Örnebring, 2016; Binns, 2017; Chen et al., 2018; Obermaier et al., 2018; Hess and Waller, 2020; Holton et al., 2021) and leads to structural issues, as many journalists adopt self-censorship strategies to avoid exposure (Löfgren Nilsson and Örnebring, 2016; Binns, 2017; Hess and Waller, 2020; Scaramuzzino, 2020). One of the most significant discussions is whether journalism's demonization affects core democratic values, such as the free expression of ideas in public debate. In this regard, many scholars argue that online harassment of journalists threatens democracy (e.g., Löfgren Nilsson and Örnebring, 2016; Waisbord, 2020; Posetti et al., 2021).

Despite this, there is a shortage of research investigating the legal system's^1^ practice and approach to journalists' exposure to illegal forms of online harassment. In the same way, few studies have examined how journalists perceive the availability of legal protections. There are, however, some exceptions that approach the topic from a peripheral perspective. For instance, a study by Obermaier et al. (2018) shows that journalists in Germany rarely take legal action when coping with online harassment. The authors hint that the predominance of emotion-focused coping, rather than problem-focused coping (for instance, by seeking legal or other professional support), may result from journalists' belief that their chances of preventing harassment are limited. Other studies have suggested that even though emotional coping strategies may provide temporary relief, they are more likely to—over the long run—result in feelings of anger, stress, and anxiety (Ferrier and Graud-Patkar, 2018; Holton et al., 2021). A few reports have also been published in Sweden that superficially addresses journalists' propensity to pursue criminal charges (Löfgren Nilsson, 2013, 2017). In these reports, it becomes clear that there is a low tendency among journalists to report online offenses. In a recent proposal for enhanced criminal protection for crimes against people exercising their freedom of expression in the context of professionally-conducted news reporting or other journalistic activities (Wegerstad, 2022, p. 2), the reluctance to seek legal support was attributed to journalists' negative experiences with the legal system and a tradition of tolerating hate and abuse within the profession. These indications conform to studies that demonstrate the normalization of harassment and abuse within journalism (Adams, 2018; Lewis et al., 2020). In addition, North (2016) shows in his study that the low level of reporting is related to fears of retaliation. However, no studies have comprehensively analyzed the perceptions behind the alleged low levels of trust in the legal system's protective abilities. Similarly, no research has examined the factors that may increase journalists' propensity to seek legal assistance in the event of illegal online harassment.

However, some studies outside the journalistic field have attempted to explain why there are so few reported cases of illegal online harassment. Specifically, this research suggests how society sanctions (both formally and informally) criminal and inappropriate behavior online in ways that differ fundamentally from that in the physical world. Abuse and harassment have, for instance, been described as a “normal” feature of the internet (Schmid et al., 2022). The trivialization of online harassment is also described as inherent in the legal system. As Citron (2014) argues, there is a tendency to blame victims for their predicaments and exposure (see also Citron and Penney, 2018). Cultural views have, in other words, been identified as a leading reason why victims do not report illegal online harassment. Other related factors are perceptions of law enforcement's technical inability, lack of resources, and expertise (Holt and Lee, 2019; Koziarski and Lee, 2020). It has also been documented that the motivation to investigate cyber-related crimes is often low among law enforcement officers (cf. Dodge and Burruss, 2019).

In terms of legal protection, many scholars agree that the legal system does not adequately protect victims of online harassment and that criminal laws pertaining to online harassment and abuse should be utilized more efficiently (Burnap and Williams, 2015; Brown, 2017; Yar, 2018; Alkiviadou, 2019). I would claim that protecting journalists' free speech is essential, especially given the flourishing post-trust tendencies and emerging populism in democratic countries worldwide. Similar arguments have, for instance, been made by Waisbord (2020), who argues that bottom-up vigilantism—driven by populist leaders—that aims to discipline and silence journalists in the public debate should not be narrowly seen as a safety problem and a matter of personal risk for journalists. Instead, it is a speech issue with substantial implications for journalism (Waisbord, 2020). Several other scholars support this claim by connecting the aim of populist movements to control of public discourse by attacking facts, news, and information with the increased demonization of journalism (Barlow and Awan, 2016; Meza et al., 2018; Sobieraj et al., 2020; Carlson et al., 2021; Posetti et al., 2021).

Given the societal importance of impartial journalism and the state's role in maintaining journalistic autonomy, it is imperative to understand how journalists perceive the legal system as a source of support and justice. The present study, therefore, contributes to the current knowledge gap by analyzing over 1,000 text responses to an open-ended survey question concerning Swedish journalists' perceptions of legal conditions in the event of illegal online harassment. The text responses were, in turn, categorized based on answers to a multiple-choice question regarding the extent to which respondents believed the legal system would protect their interests when exposed to online harassment. As a result of this method, perceptions of respondents with varying levels of trust in the legal system are examined. The following research question is addressed in this paper:

- How do Swedish journalists perceive legal conditions in the event of online harassment?

This study seeks to investigate journalists' perception of and trust in the legal system as a unified construct rather than examining individual legal norms.^2^ This research explicitly examines journalists' perception of their access to legal aid, referred to in this study as legal conditions. Consequently, by responding to the research question, this study contributes to the existing body of literature by investigating journalists' perceptions of legal protection. It also provides insights into the basis of such perceptions. Although conducted within a Swedish context, this study has implications for the international scientific community, due to the increasing prevalence of online harassment against journalists in western democracies (Fadnes et al., 2019). It should be noted, however, that Swedish and European legislation differs from that of, for example, US law. Nonetheless, as the primary focus of this study is not legal in nature, the findings are of international significance. Apart from adding scientific value, the results can also be relevant for governments and civil society organizations seeking to provide means and information to protect journalists from the harms associated with online harassment.

## Method and materials

Two survey questions were used in this study: (1) a multiple-choice question, and (2) an open-ended question. While data from the open-ended question formed the basis of the qualitative analysis, the multiple-choice question served as a methodological tool for categorizing the extensive qualitative data concerning respondents' trust in the legal system.

### Participants

Following approval from the Swedish Ethical Review Authority, an online survey was administered between December 17, 2020, and January 14, 2021. The survey was sent to 9,603 members of the Swedish Union of Journalists and collected ~3,000 responses. Participants were notified by email about the study 1 week prior to the launch of the survey. In this information email, they were instructed not to participate if their work did not expose them to the risk of online harassment. On the first page of the survey, the same statement was repeated. Although there is no available assured data, it is reasonable to assume, based on previous studies and reports (cf. Hedman, 2016; Ekberg et al., 2018), that the number of journalists in Sweden who are at risk of exposure to online harassment due to their job assignments is in the range of 3,000–6,000.

The respondents were evenly distributed in terms of gender (female: 1,649; male: 1,384) and age.^3^ Regarding geographical demographics, respondents were mainly concentrated in the three largest cities in Sweden (Stockholm, Gothenburg, and Malmö); particularly in Stockholm. A similar geographical distribution has been demonstrated in branch-mapping reports (Ekberg et al., 2018).

### Survey procedure

As journalism involves writing, respondents were expected to provide more and lengthier responses than other research populations. The idea was that by using an open-ended question, this study would capture diverse and alternative views (Popping, 2015; Zhou et al., 2017) regarding journalists' perceptions of available legal aid in the event of online harassment. In addition, depth of the responses is further enhanced by adding the multiple-choice question, which categorizes the level of trust in the legal system's ability to provide adequate support in such a scenario.

It is crucial to keep in mind, however, that certain factors may impact respondent's answers or willingness to submit a response to open-ended questions. Some research suggests that respondents' responses to multiple-choice questions may significantly predict their responses to any open-ended questions that follow. Research has, for instance, shown that respondents who provide responses indicating dissatisfaction to multiple choice questions more often reply to open-ended follow-up questions and provide longer answers about situations that elicit negative feelings (Poncheri et al., 2008; Borg and Zuell, 2012). There is, nevertheless, some controversy surrounding such findings, as other studies have found no correlation between responses to close-ended and open-ended questions (Zuell et al., 2015).

### Survey questions

The multiple-choice question was formulated as follows: *Imagine that you recently, in connection with your role as a journalist, have been exposed to any of the crimes just described*.^4^ *To what extent do you think the judicial system has the ability to take care of your interests as a crime victim?* This question was answered on a five-point Likert-like scale (1 = very good ability, to 5 = very poor ability). Following the multiple-choice question, participants were asked to elaborate on their answers in the open-ended question: *Feel free to develop your views on the legal conditions for journalists subjected to threats and hatred*.^5^

### Data management and analytical model

The participant's responses to the multiple-choice survey question were analyzed using descriptive statistics, and the text responses (open-ended questions) were categorized based on the five answer options in the multiple-choice question: very good ability, fairly good ability, neither good nor bad ability, fairly poor ability, and very poor ability (see Table 1).

**Table 1:** Respondents' perception of the legal system's ability to safeguard their interests as crime victims in the case of illegal online harassment (multiple-choice question), and information regarding the text responses (TR) to the open-ended question.

	**Frequency (MCQ)**	**Percent (MCQ)**	**Frequency (TR)**	**Percent (TR)**	**Word count (TR)**	**Avg. word count (TR)**
(1) Very good ability	49	1.7%	18	36.7%	598	33
(1) Fairly good ability	523	18.4%	140	26.7%	4,358	31
(2) Neither good nor bad ability	816	28.8%	197	24.1%	7,083	36
(3) Fairly poor ability	1,084	38.2%	437	40.4%	13,823	32
(3) Very poor ability	365	12.9%	188	51.5%	7,136	38
Total	2,837	100%	980	34.5%	32,998	33.6

System missing for the multiple-choice question (MC) = 257. Percent text response (TR) represents the percentage of open-ended responses relative to the frequency of multiple-choice responses. Avg. word count (TR) represents the average word count per response to the open-ended question. The numbers (1, 2, and 3) next to the Likert-like scale answers represent the three categories used in the results section. (1) High trust in the legal system's protective abilities; (2) Neither high nor low trust in the protective abilities of the legal system; and (3) Low trust in the protective abilities of the legal system.

As illustrated in Table 1, 51% of respondents assessed the legal system's protective abilities as either “fairly poor” (38.2%) or as “very poor” (12.9%); 28.8% assessed their abilities as “neither good nor bad;” 18.4% rated the legal system as “fairly good” and only 1.7% rated it as “very good.”

Table 1 demonstrates general disbelief in the judicial system's ability to protect journalists exposed to online harassment. In other words, the results indicate that many journalists have low trust in the legal system. Furthermore, a significant proportion of respondents view the legal system's protection as neutral. However, the quantitative data provide limited insight into journalists' perceptions. For example, it may be beneficial to understand why some journalists view the legal system's abilities as adequate or favorable despite the low percentage displayed in Table 1. In order to gain such insights, qualitative text analysis has been conducted on the text responses associated with the answer options in Table 1.

Qualitative text analysis was carried out in three steps (Bernard et al., 2016): (1) observation of recurrences and repetitions within the data; (2) search for similarities and differences by making systematic comparisons across data units; and (3), identification and selection of illustrative quotes representing the various themes. The first step involved a review of a random sample of responses from each level of trust to identify possible key themes (Sandelowski, 1995). At this initial proofreading stage, recurrences and repetitions were noted. Recurrences were observed in the form of similar sentiments expressed differently, as opposed to repetitions arising directly from similar written expressions (Owen, 1984). The more often a perception was expressed, the more likely it was to be observed as a theme. The second step involved searching for similarities and differences by systematically comparing data units. This approach was inspired by Glaser and Strauss's (1967) “constant comparison method,” which encourages line-by-line analysis by asking: “what is the sentence about?” “how is it similar or different from the preceding or following statements?” and “is there any difference in the degree or kind to which the theme is articulated?” By constantly questioning the meaning of the responses, different subthemes and nuances emerged, as this analytical model forced comparisons. Finally, quotes representing the essence of various themes were identified and arranged in the third step. In the next section, I will present the principal findings of the current study.

## Results

As a means of improving the readability of the results, the five answer options were reduced to three categories: (1) high trust in the legal system's protective abilities, (2) neither high nor low trust in the protective abilities of the legal system, and (3) low trust in the protective abilities of the legal system. The text responses from the participants who answered: “very good abilities” and “fairly good abilities” are thus compounded into one category; the same applies to the responses from the respondents who answered: “very poor abilities” and “fairly poor abilities.” For the second category, 24 text responses expressed in different ways that they had no opinion; presumably because the question did not include an “I don't know” option. These 24 text responses were removed from the analysis.

As shown in Table 1, respondents who have a low level of trust in the legal system submitted more open-ended responses than respondents in the other two categories. Similarly, respondents who assessed the legal system's ability to provide protection as “very poor” used more words per answer (38). As discussed in the method section, this is consistent with research showing that respondents who provide unsatisfactory responses are more likely to reply to open-ended follow-up questions and write longer responses.

Quotes in the results section have been translated from Swedish into English. As a result of structural differences between the two languages, some grammatical adjustments have been made. These grammatical changes do not affect the sentiment expressed in the text responses. Each quote is supplemented by a footnote with information about the respondent's gender, age, professional role, workplace outlet, and experience of online harassment.^6^ Most quotations have been left intact, except for a few longer quotations that have been shortened to omit irrelevant information. Each quote represents an open answer from a unique respondent.

### Perceptions among journalists with high trust in the legal system's protective abilities

In recent years, the online harassment of journalists has been widely discussed in Swedish public discourse as a possible threat to democracy. As a result, measures have been taken to counter this development. Specialized democracy and hate crime units have, for instance, been established in the three metropolitan police regions to investigate crimes against journalists and politicians, among others. Furthermore, there have been reports in the media of convictions against individuals who have threatened journalists. Those who trust in the legal system's abilities have noted these developments, concluding that the legal circumstances have improved:

“*My feeling is that more people are reporting and that there have actually been convictions in some cases. So, if this happens to me in the near future, I want to believe in the legal system.”*^7^

“*The conditions have improved, I was regularly subjected to threats 10 years ago, and then the police shrugged their shoulders.”*^8^

These beliefs assume that the legal system will aid and support journalists asking for help. While it is difficult to assess whether these perceptions are based on prior contact with the legal system, they nevertheless suggest a willingness to use the legal system if it is thought to lead to just treatment and potential convictions.

It emerged, however, that some respondents had interacted with the legal system after being harassed or threatened. From these responses, it became evident that a successful legal outcome had a positive effect on their experiences and, consequently, their trust in the judicial system. Interestingly, being treated with dignity, respect, and competence was just as—if not more—influential on respondents' perceptions of the legal system's efficacy. It was acknowledged that it could be difficult for the legal system to achieve a successful conviction. Nevertheless, respondents maintained a positive outlook on the legal system due to the treatment they had received. The following quotes illustrate these perceptions and experiences:

“*About 4 years ago, I was threatened and jumped on by a gang that is now fairly well-known as ‘the clan in Angered.'*^9^ *The police immediately took up the matter and the unit for ‘crimes against democracy' carried out a serious and good investigation.”*^10^

“*The police have taken both my personal reports as well as reports concerning employees seriously, which has also happened in the continuing legal chain in cases where it has proceeded to prosecution.”*^11^

These results indicate that favorable treatment from law enforcement officers is critical in fostering trust in the legal system. This is because it can help to create a sense of security and an atmosphere where journalists feel at ease when engaging with law enforcement officers. Moreover, it bolsters confidence in the legal system and cultivates a sense of mutual respect between journalists and actors within the legal system.

An additional factor contributing to high trust in the legal system was the perception that it was relatively straightforward to collect digital evidence. Respondents who expressed this sentiment thought that print screens of threatening messages or the sender's IP address would make up a substantial portion of the evidence presented in court. It was a widely-held belief that if such evidence were collected, there would be a heightened likelihood of a conviction. One respondent emphasized this point while also stressing the possibility of obtaining legal redress for “milder” offenses.

“*It is no different from anyone else regarding the ability to prove that a crime has been committed. If there is supporting evidence, the legal system can convict people for the sometimes more subtle crimes of molestation, sexual molestation, and threats. Regardless of whether you are a journalist or not. I have sat in on hundreds of trials and have therefore seen what many do not believe: convicting people for these crimes is perfectly possible. If the evidence is sufficient, that is.”*^12^

Contrary to previous studies which have suggested that people generally perceive the legal system as being unable to resolve crimes in online environments due to the complexity of the technical aspects and the lack of resources and knowledge (cf. Holt and Lee, 2019; Koziarski and Lee, 2020), this finding reveals that some participants attribute the digital nature of the crimes to be an advantage relative to the burden of proof. Recent campaigns conducted in Sweden concerning how to save evidence when exposed to online harassment may explain this result. However, it should be noted that there were a few participants who held the opposite opinion. Those participants stated that, despite having faith in the legal conditions, securing evidence was one of the main challenges, particularly when perpetrators were anonymous. Such beliefs are more prevalent among individuals with low trust in the legal system, as will be discussed further on in the results section.

Despite their optimism regarding the legal system's capabilities, respondents in the first category also expressed concerns regarding matters that do not fall within the scope of the law. For instance, it was stressed that people who voice more serious threats conceal their identities or express themselves aggressively, yet legally. Related to this, several respondents also found it difficult to determine whether an aggressive or hateful message was unlawful. It was likewise noted that harassment and hate affect individuals differently, and that the legal system lacks a comprehensive understanding of the harm caused by crimes with low penalties. Two short answers illustrate this point:

“*I think it varies a lot from person to person on how seriously you take these crimes and what level of ambition you have when it comes to solving them.”*^13^

“*The challenge is that it is a matter of judgment. What one experiences as an offense is very personal and the judicial system can make other evaluations and assessments.”*^14^

These results suggest that increased hostility toward journalism can be attributed to a form of discursive violence that is often challenging to regulate through legal means. Waisbord (2020) has previously made a similar argument, highlighting that online harassment is driven by a form of virtual violence that operates within the boundaries of the law to assert power over journalists. Even when sufficient legal conditions are present in certain well-defined situations that are accompanied by compelling evidence, the more pressing concern can be the sheer volume of minor harassment that, compiled, can reduce journalists' sense of safety. Considering this, it is interesting to note that both the above quotes are from journalists who reported having no experience of online harassment in the last 3 years.

In relation to matters that fall beyond the purview of the law, some respondents suggested that journalists, in comparison to the general public, might be less likely to turn to the legal system when subjected to unlawful harassment. This opinion was based on the notion that journalists are expected to cope with animosity and intimidation as part of their profession. For example, one respondent commented that, although they thought legal remedies were accessible, the legal system predominantly serves the interest of individuals and not journalists:

“*They [the legal conditions] seem good on paper. But most of the time I choose not to report it, since as a public figure you have to endure and count on some attacks. I got the idea that the legal system is primarily for private individuals who are violated and not professional journalists. But you should report it—at the same time it feels as if that is exactly what those who spread the information want you to do.”*^15^

Moreover, it was emphasized that employers had become more aware of the negative effects of online harassment, which was viewed as a prerequisite for bringing legal action. However, as one participant pointed out, this dynamic is complex and may clash with journalists' professional image, preventing many from contacting law enforcement:

“*If we only dare to report and get support from our bosses to do so, I hope and believe that we have the same rights as everyone else. But I think the difficult part is to see yourself as the ‘victim' you are and balance the power you have as a journalist; that is, people's attitude toward journalists is partly connected to the fact that we are a type of power holder.”*^16^

On the other hand, it was also stressed that the issue is primarily attributed to how employers manage harassment as opposed to the legal system:

“*I have reported threatening messages in the past and was surprised that the police seemed to take it very seriously. In the end, the investigation was dropped, it was an abstract threat, but my strongest impression was rather that employers are not fully prepared for that type of event anymore.”*^17^

There is a clear divergence between these outcomes and those previously reported in the first category. Instead of indicating a lack of confidence in the legal system, these responses imply that journalists may be hesitant to seek legal assistance due to cultural norms within the profession. Under the results section, these findings will be discussed and analyzed in more detail.

### Perceptions among journalists with neither high nor low trust in the protective abilities of the legal system

The perceptions in categories one and two partially overlapped. However, respondents in the second category were more likely to be critical of the legal system. It was highlighted that addressing their exposure to harassment can be tricky, since harassment is often expressed ambiguously, making it difficult to decide whether it is illegal. Many argued that the legal system needs to improve its ability to handle less severe harassment with more appropriate sanctions, and that there is a need to clarify what is considered illegal online harassment. It was often noted that for most harassment cases, there was not much in the way of available recourse, leaving journalists to deal with the negative aspects of exposure on their own. Respondents were critical, expressing that the legal system only seemed to act when the threats were explicit and egregious, as highlighted in the following response:

“*It seems from the outside that there has to be a very high level of threats and hatred and that it will take a long time for it to become a legal case at all… ‘ordinary' harassment seems to be of little interest to the legal system.”*^18^

At initial inspection, the above quotations suggest a definitive conclusion: no legal action can be pursued without sufficient evidence. However, these results also allude to how journalists perceive the judicial system's attitude toward online harassment. Generally, it is believed that the legal system does not adequately acknowledge the detriment that online harassment has on journalists' capacity to perform their job. The precise rationale for this is beyond the scope of this article; yet, as prior studies have revealed (e.g., Dodge and Burruss, 2019), motivation to investigate cybercrimes is often low among law enforcement officers, which resonates with these results.

It is suggested that there is a need for increased clarity regarding how the legal system handles online harassment. There is a similar consensus concerning employers' approaches to journalists' exposure to online harassment. Although this research focuses on the perceptions of journalists concerning the legal system, employers have a fundamental part to play in generating a secure workplace, including offering help to journalists before and during potential legal proceedings (Holton et al., 2021). Unfortunately, these concerns are not being addressed systematically, as one respondent pointed out:

“*In addition to the fact that there is hatred and threats against journalists, a big problem is that this is hardly discussed in newsrooms or in journalism education programs. There should be a well-thought-out plan for how editorial management should deal with the issues—at all editorial offices. And all violations should be reported to the police.”*^19^

According to several respondents, harassment is so prevalent that it has become normalized as a part of the occupation. It is probable that the unsystematic work environment approach from news organizations partially observed in the results contributes to this normalization. This lack of perceived legal protection and the recurring and habitual nature of abuse is a cause for concern. Interestingly, it was proposed that these two elements could be interrelated:

“*For many journalists, it is a part of professional everyday life that has become more and more tangible in step with increasingly faster and simpler forms of technical platforms for communication. Historically, there is probably also a discursive acceptance of threats and hatred as part of the professional role, which certainly contributes to sluggishness in the development of the view of hatred and threats against journalists and the shaping of an effective legal system in this area.”*^20^

As demonstrated by the quote above, “the discursive acceptance of threats and hatred” can be seen as a contributing cause as to why journalists are hesitant to report online harassment. This is a clear example of how law and norms do not function in concert to protect the speech of journalists. In other words, professional norms instruct journalists on “appropriate” and “inappropriate” ways of dealing with harassment. When these messages are internalized, it can become challenging to show oneself as being vulnerable to exposure, and hence even more difficult to take the step to seek legal support. Moreover, if it is perceived that the legal system shares values suggesting that journalists should be able to deal with harassment on their own, the uphill climb becomes even steeper.

### Perceptions among journalists with low trust in the legal system's protective abilities

The most pervasive perception among respondents with low trust in the legal system is that online crimes against journalists are not adequately prioritized. In contrast to those with high trust in the legal system's abilities—which is often attributed to positive interactions with police and prosecutors—negative experiences are often the cause of low trust. For example, many believe that filing a police report is futile, as the charges are likely to be dismissed, regardless of the strength of the evidence presented. Therefore, negative experiences with the legal system or information obtained from colleagues with negative experiences have led many to refrain from contacting police when exposed to online harassment. The following quotes exemplify the experiences of two respondents who have sought out the legal system after being exposed to, in their opinion, severe forms of harassment:

“*I have experienced that a preliminary investigation was dropped when I received death threats because of my work, despite recordings and other evidence with the motivation that ‘we don't make that kind of an effort for a threatened journalist'.”*^21^

“*I have, among other things, received messages that I considered contained clear molestation or sexual harassment. This was in connection with my journalistic work. On one occasion, I reported to the police when, in my opinion, the whole thing became quite rough and had happened repeatedly from the same person. But the case was dropped immediately. Sad, when both the person who received the report on the phone and I spent much time describing and writing down what had happened.”*^22^

These findings further bolster the patterns observed in the preceding category; namely that many journalists perceive the legal system to be dismissive of online harassment as a criminal offense. Consequently, many journalists are doubtful that legal actors will adequately fulfill their obligations. There is compelling evidence that journalists do not feel they are being accorded fairness by the legal system, thus conveying the impression that their grievances are of no importance to them.

Another noteworthy discovery uncovered that it was commonly believed that the legal system lacked the skills and resources to examine digital transgressions. These impressions can likewise be ascribed to the lack of priority given to these cases. While a few respondents briefly mentioned technological difficulties in investigating these crimes—for example, crimes associated with anonymous senders—the larger part of responses shows that the essential issue lies in a lack of technical expertise on the part of the legal system. As one respondent commented:

“*It is unacceptable that things that would be obvious crimes if communicated face to face are not sanctioned. It is clear that the legislation is not being followed*—*and it does not seem to be a question of resources but of the legal system's competence. The legal system cannot prove crimes, and they do not invest resources in hunting down anonymous online trolls. Which they should. If they do not have the skills, they have to get them because this is not something that will decrease or disappear. However, you can report via social media, this usually leads to accounts being closed.”*^23^

It is noteworthy that this result does not pertain to a legal matter; very few, if any, respondents complained about the existing legal framework. Instead, a more pressing issue is how the legal system approaches digital crimes (living law) and the distinction between digital offenses and “real-world” offenses. As one respondent succinctly summed up: “if no blood, no resources.” This highlights the apparent lack of effective pathways of protection and justice from the harms associated with digital offenses. This observation is corroborated by other studies demonstrating that journalists dealing with exposure to illegal online harassment tend to rely on individual strategies instead of turning to the law for justice (Ferrier and Graud-Patkar, 2018; Holton et al., 2021).

In accordance with these findings, several respondents highlighted that certain actors within the legal system viewed online harassment as an unavoidable aspect of a journalist's job. Consequently, from the perspective of these participants, it was assumed that journalists should be able to tackle these issues autonomously, without the support of the state. The following two quotes illustrate this:

“*A general and problematic attitude among police is that journalism generates ‘lifestyle crimes' where the person subjected to crime is thus considered an accomplice. A profession as a journalist means an increased risk of being exposed to crime. With that, you are considered to have deliberately exposed yourself to increased criminal acts that can be directed against you.”*^24^

“*The police and law enforcement authorities show a demonstrative disinterest aimed at making the victim understand that the crimes we are subjected to are of no interest to them, alternatively (in my darkest moments of vulnerability) that they sympathize with the political circles (Sweden Democrats) that organize the crime and the terror.”*^25^

As these quotes indicate, many respondents perceive that attacking journalists is socially acceptable and that society sanctions such attacks. Thus, this echoes the sense that exposure is “a part of the game.” The perceptions of these respondents are further strengthened by the fact that they have been heavily exposed (see footnotes 24 and 25) to online harassment in the last 3 years.

The remarkable finding that emerged from the lack of clear legal protection for journalists was that many journalists were apprehensive about pressing charges, as they felt that this would only exacerbate the situation. This apprehension was based on the belief that by taking action and filing a complaint, they would be tacitly admitting to feeling intimidated and threatened by the harasser, thus inadvertently giving the harasser a “victory.” These sentiments were elucidated in the responses, such as in the following quote:

“*The basic problem with threats is that you as a journalist do not want to show that you are afraid*—*then the perpetrator has succeeded and may be inspired to continue or expand the threats. I quit as a crime reporter after many years of intimidation, but in the past, I often wrestled with that dilemma. It was about playing non-chalant when gang members tried to psych you out. In order for a report to lead to a conviction, it is required that I tell you in detail about my fears, nightmares, and what I fear will happen to my children. I don't want to give them that…”*^26^

Another respondent went on to explain how their workplace security division had even taken to mediating such issues, further demonstrating the passive attitude that many journalists take toward harassment:

“*Many of those who make threats/hate do not care about the police. The job's security officer often advises against reporting to the police. Most recently, I myself wanted to abstain, as I did not want more attention in other media. The police naturally find it difficult to follow up on threats/hate, and then there is zero interest in entering into such a process. The risk is rather that you fire on those who persecute you and get no help. It is not possible to get protection 24/7 for a long time. Better to hope it goes away.”*^27^

This is indicative of a pervasive culture of passivity when it comes to responding to harassment within the profession. Consequently, it is essential for journalists to be provided with adequate legal protection and support to ensure that their rights are upheld and that they can act in the event of illegal harassment. Only then will journalists have the confidence to hold perpetrators accountable. Based on these results, it is evident that the lack of clarity surrounding support from the legal system and cultural patterns within the profession that diminish the severity of harassment create a difficult environment for journalists to display vulnerability. As the quotes above demonstrate, respondents are adversely impacted by the threats they encounter. The legal system or security officers do not address these issues, thus leading to an inaction. As the results demonstrate, journalists are conscious of this passive attitude. This cognizance then affects the perceived possibilities for action and access to justice. Consequently, if journalists believe they have no options in dealing with online harassment, it is reasonable to assume that many suppress the negative emotions associated with exposure.

Furthermore, respondents with low trust in the legal system asserted that the perceived lack of legal safeguards, combined with the magnitude and frequency of exposure, has deleterious consequences for individual journalists and society alike. Several of the responses reiterated previously-discussed themes in the third category, while adding greater urgency to the harmful implications. For example, it was stated that the current climate necessitates many modify their work in response to hostile discourse. As illustrated in the comments below:

“*Freedom of expression is and should be extensive and far-reaching. But at the same time, threats and hatred*—*above all the kind that are not crystal-clear illegal*—*make people adapt, perhaps unconsciously, to choose other subjects, other angles, etc. In the long run, of course, a democratic problem. Or that more and more people cannot bear to remain in the profession.”*^28^

“*I feel that there is very little ability and willingness to help those who are exposed. There are too few convictions for online hate and threats, it is too easy to email anonymously and threaten, and too many cases are closed. There is a certain category of people who know exactly how far they can go and who also do [so]. In recent years I have chosen to keep a much lower profile because of this and because I have been threatened in the past because I have an unusual name and also have small children.”*^29^

“*Precisely because this is a development that has been going on for a long time, for the past 3 years, I have more or less gone ‘under the radar' and refrained from including my name in bylines or participating in contexts (e.g., some panels and debates) in order to avoid reactions as hate/threat. If the questions had been asked 7–8 years ago, the answers would have been different.”*^30^

The findings of this study provide insight into the detrimental consequences of online harassment against journalists, highlighting how the legal system is not equipped to handle such matters appropriately. These responses suggest that journalism's autonomy is compromised by the speech expectations accompanying increased hostility toward journalism. Consequently, it is questionable whether the legal system is adequately prepared to manage online harassment's chilling effects on journalism. Furthermore, as Penney (2021) argues, chilling effects not only involve a deterrent effect, but also a shaping effect, in which people adhere to perceived social norms; or in this case, the regulation of speech by hateful actors through informal means. These claims, however, exceed the empirical scope of this study. Nevertheless, it raises concerns about the legal system's current role in protecting journalists' speech and autonomy.

## Discussion and conclusions

This study set out to investigate how Swedish journalists perceive legal conditions in the event of online harassment by analyzing open-ended text responses from survey respondents who had varying levels of trust in the legal system. The most prominent finding from this study is that journalists perceive that the legal system trivializes the seriousness of exposure to online harassment. However, the results reflect several nuances contributing to a comprehensive understanding of the relationship between journalists and the legal system. In the following section, these nuances will be discussed in additional detail.

This study found that previous experiences with the legal system influenced respondents' willingness to contact law enforcement agencies in the event of exposure to unlawful online harassment. It was evident that being treated respectfully by legal system actors was as influential, if not more influential, than the outcome of the criminal complaint. In addition, several respondents with a high level of trust in the legal system attributed their beliefs to information they received from colleagues and knowledge of recently reported convictions. These findings suggest that the legal system's mediated approach to online harassment affects attitudes and norms relating to legal protection. Consequently, it reveals a unique insight into how journalists respond to the message conveyed by the legal system. Notably, this result implies that when such messages are internalized, journalists feel more empowered to take measures against online harassment. It also corroborates empirical observations by Citron and Penney (2018), which indicate that the legal system can empower victims and prevent the chilling effects of online harassment. When interpreting these results, it is critical to remember that journalists with high levels of trust in the legal system were not exposed to online harassment to the same degree as journalists with lower levels of trust (see footnotes in the result section).

Furthermore, social norms within journalism play a significant role in shaping journalists' responses to and views of online harassment. In other words, perceptions of a stigmatizing culture influence how the profession responds to online harassment, consistent with previous research underscoring the normalization of such abuse within the profession (North, 2016; Adams, 2018; Lewis et al., 2020). Interestingly, these cultural aspects have yet to be examined in relation to the protective capabilities of the legal system. The results of this study suggest that traditional discursive acceptance of threats and hatred is intertwined with how the legal system perceives and deals with online harassment. Simply put, there is a reciprocal relationship between normalizing such abuse and the legal system's commitment to addressing it.

It was generally assumed that the judicial system only addressed egregious or potentially violent forms of harassment. Consequently, it was believed that there was limited recourse for other types of harassment. This presents a challenging dilemma: while many forms of harassment may not be legally actionable, a greater proportion of these cases are likely to be deemed unlawful than journalists may be aware of. In light of this, the sense of helplessness expressed by respondents concerning formal measures should be viewed from a more comprehensive standpoint. By minimizing harassment, we reinforce the idea that it is a common element of the Internet (Schmid et al., 2022). The legitimization of harassment, both by the legal system and within journalism, demonstrates a lack of comprehension concerning the adverse psychological impacts of exposure to online harassment. This ignorance is made evident by the fact that resources and priorities are perceived to only be allocated when there is a potential risk of physical confrontation. These results are likely a consequence of online offenses having a lower status than those occurring in the physical world and therefore not receiving sufficient attention from the legal system.

In conjunction with this, journalists often lack clear avenues of support when confronted with unlawful online harassment. Due to the absence of established norms and behavior guidelines for abuse victims, they cannot rely on the same legal system as they would if the crime were to occur offline. For example, if journalists were threatened with an unlawful act in person, they may feel more confident in seeking help from the legal system. However, in the event of the same unlawful act occurring online, they do not have the same access to legal protection. As a result, it can be inferred that laws, attitudes, and norms interact weakly to empower journalists in a digital society.

Consistent with prior research (Dodge and Burruss, 2019; Holt and Lee, 2019; Koziarski and Lee, 2020), many respondents believed that the legal system does not adequately prioritize online harassment and lacks the necessary technical expertise to address it. The most frequently cited reason for these views was linked to personal experiences with the legal system. Those with low trust in the legal system often reported feeling neglected when seeking judicial assistance. These findings are particularly significant when considering how the legal system's treatment of journalists may shape expectations related to protection. Compared to the beneficial effects of fair treatment, the detrimental consequences of perceived inadequate treatment become all the more glaring. This study underscores the importance of treating journalists with dignity, courtesy, consideration, and respect for their rights to enhance their confidence in their ability to obtain protection. Conversely, a lack of such treatment can severely weaken journalists' sense of security and safety.

Having discussed the basis upon which respondents construct their perceptions, it is important to emphasize the fact that the results of this study indicate that online harassment diminishes the autonomy of Swedish journalism. Text responses indicate that journalists have avoided covering topics that they perceive to provoke hostile reactions, as well as concerns that “controversial” reports and angles may not even be considered in light of hateful online discussion climates. Additionally, the study found that respondents have taken on fewer or refused all public assignments due to a lack of will or energy to handle exposure over an extended period of time. These results point to toward a dysfunctional public debate setting, as the imminent threat of online violence and hatred discourages journalists from freely voicing various viewpoints. As such, these results also confirm and demonstrate the effects of what previous studies have already described; namely how the collective ambition to demonize professional journalism effectively suppresses journalistic voices (cf. Barlow and Awan, 2016; Meza et al., 2018; Sobieraj et al., 2020; Carlson et al., 2021; Posetti et al., 2021). Furthermore, this study indicates that the legal system is inadequate in countering the adverse control that online harassment collectively constitutes. Although this is a preliminary finding, it suggests that the perceived absence of legal protection partly shapes journalists in normalizing harassment. At the very least, it signals that it is nearly impossible to formally intervene through legal venues.

In conclusion, this study provides a novel insight into Swedish journalists' perceptions of legal conditions in relation to online harassment. The findings of this research form the basis for further exploration of the relationship between journalism, norms, and the legal system to protect journalists' autonomy in the digital era. It is evident from the results that further efforts should be taken to increase the availability of legal assistance to journalists. Nonetheless, it is essential to emphasize that online harassment is not just an issue of the law but a broader social concern that touches upon morality, human respect, and democratic values. In this regard, this study emphasizes the necessity for a comprehensive strategic approach to overcoming the absence of norms and behavior guidelines for journalists subjected to online abuse. This includes initiatives targeted at legal system actors, journalist employers, and journalists. Such strategies should, in other words, consider criminal law, the psychological and social ramifications of exposure, and the need to challenge the normalization of harassment in the journalistic profession. For instance, employers should address less conspicuous forms of harassment as a psychosocial work environment problem instead of not strictly viewing it as a potential security threat. The result of this study indicates a need for a more proactive and organized approach from news organizations on this matter. Furthermore, legal resources should be made available to enable more successful law enforcement concerning both law in the books and law in action. In particular, there is a need to raise awareness of journalists' vulnerability and the effects of online harassment on democratic values within the legal system.

## Data availability statement

The raw data supporting the conclusions of this article will be made available by the authors, without undue reservation.

## Ethics statement

The studies involving human participants were approved by the Swedish Ethical Review Authority. The participants provided their written informed consent to participate in this study.

## Author contributions

The author confirms being the sole contributor of this work and has approved it for publication.

## Footnotes

^1^The term “the legal system” is employed throughout this paper as a reference to law enforcement (police or any other enforcement agency) and the criminal justice system (for instance, courts, and prosecutors).

^2^This approach resembles procedural justice, where the focus often lies on how people perceive the fairness and legitimacy of legal systems and how this, in turn, affects trust (Sunshine and Tyler, 2003; Tyler and Blader, 2013).

^3^*N* = 18–25 years: 79; 26–35 years: 573; 36–45: 681; 46–55: 892; 56–65: 693; older than 65: 124.

^4^Before answering this question, respondents were asked how frequently they had experienced different forms of illegal harassment over the last 3 years. In more precise terms, this section comprised a series of short descriptions, inspired by The Swedish Internet Foundation, explaining the legal definition of commonly-occurring internet-related crimes of relevance to this study. Seven crimes were outlined in the survey: defamation, insulting conduct, unlawful threat, molestation, sexual harassment, hate speech, and unlawful invasion of privacy.

^5^The expression “hatred and threats” is a direct translation of the Swedish formulation from the survey, often used in the Swedish context. “Online harassment” is the English equivalent.

^6^Regarding exposure to online harassment, I distinguish between milder forms of harassment and more severe forms of harassment following the Swedish penalty scale. In this study, milder forms of harassment refer to insulting conduct, defamation, and molestation, while more severe forms of harassment refer to unlawful threats, sexual harassment, and hate speech.

^7^Male publisher/columnist in his late 20's to early 30's who works for a daily newspaper in a small city. During the last 3 years, he has occasionally been exposed to milder forms of online harassment.

^8^Male columnist in his late 30's to early 40's who works for a weekly/monthly magazine in a small city. During the last 3 years, he has occasionally been exposed to milder forms of online harassment.

^9^Angered is a neighborhood in the northeastern region of Gothenburg.

^10^Stockholm-based reporter in his late 20's to early 30's who works for a daily newspaper. During the last 3 years, he has often been exposed to milder forms of online harassment and, on occasion, more severe forms of online harassment.

^11^Female publisher, columnist, and news director in her late 40's to early 50's who works for a daily newspaper in a mid-sized city. During the last 3 years, she has often been exposed to milder forms of harassment and, on occasion, more severe forms of harassment.

^12^Female reporter in her late 50's to early 60's who works for a daily newspaper in a mid-sized city. During the last 3 years, she has been exposed to mild forms of online harassment in a few instances.

^13^Female reporter in her late 30's to early 40's who works for a daily newspaper in a mid-sized city. During the last 3 years, she has not been exposed to online harassment.

^14^Male freelance journalist in his late 50's to early 60's who works in a mid-size city. During the last 3 years, he has not been exposed to online harassment.

^15^Stockholm-based editor-in-chief, publisher, and reporter in his late 30's to early 40's who works for an online newspaper. During the last 3 years, he has been exposed to milder forms of harassments very often and, on occasion, to more severe forms of harassment.

^16^Stockholm-based reporter in her late 30's to early 40's who works for a union newspaper. During the last 3 years, she has occasionally been exposed to milder forms of online harassment.

^17^Stockholm-based columnist/photographer in his late 20's to early 30's who works for a news agency. During the last 3 years, he has occasionally been exposed to milder forms of online harassment.

^18^Male radio program host in his late 40's to early 50's who works in a mid-size city. During the last 3 years, he has occasionally been exposed to milder forms of online harassment.

^19^Female freelance journalist in her late 30's to early 40's who works in a mid-sized city. During the last 3 years, she has, on a few occurrences, been exposed to online sexual harassment.

^20^Male editor and writer in his late 40's to early 50's who works for a weekly/monthly magazine in a mid-size city. During the last 3 years, he has not been exposed to online harassment.

^21^Stockholm-based reporter in her late 30's to early 40's who works for an evening paper. During the last 3 years, she has occasionally been exposed to milder forms of online harassment.

^22^Malmo-based freelance journalist in her late 40's to early 50's. During the last 3 years, she has occasionally been exposed to milder and more severe forms of online harassment.

^23^Stockholm-based reporter and columnist in her late 40's to early 50's who works for a daily newspaper. During the last 3 years, she has occasionally been exposed to milder forms of online harassment and, in a few instances, to more severe forms of online harassment.

^24^Stockholm-based freelance journalist in his late 50's to early 60's. During the last 3 years, he has been exposed to milder and more severe forms of online harassment very often.

^25^Stockholm-based freelance journalist in his late 40's to early 50's. During the last 3 years, he has been exposed to milder and more severe forms of online harassment very often.

^26^Stockholm-based reporter and columnist in his late 40's to early 50's who works for a daily newspaper and television station. During the last 3 years, he has been exposed to milder forms of online harassment very often and to more severe forms of online harassment often.

^27^Stockholm-based reporter and columnist in his late 40's to early 50's who works for a daily newspaper and television. During the last 3 years, he has very often been exposed to milder forms of online harassment and often to more severe forms of online harassment.

^28^Gothenburg-based program host on a radio station in her late 30's to early forties. During the last 3 years, she has occasionally been exposed to milder forms of online harassment.

^29^Stockholm-based reporter, columnist, and program host in her late 50's to early 60's currently between jobs. During the last 3 years, she has been exposed to milder forms of online harassment often, and occasionally to more severe forms of online harassment.

^30^Stockholm-based television program host in her late 30's to early 40's. During the last 3 years, she has occasionally been exposed to milder forms of online harassment, and in a few instances, to more severe forms of online harassment.
